# Physical exercise and crisis coping in college students: evidence from a chain mediation model of resilience and emotion regulation

**DOI:** 10.3389/fpsyg.2026.1762870

**Published:** 2026-05-11

**Authors:** Mengzhe Song, Qifeng Sun, Jianda Li, Shimeng Wang, Bochun Lu

**Affiliations:** Institute of Sports Science, Nantong University, Nantong, China

**Keywords:** cognitive reappraisal, college students, crisis coping, emotion regulation, physical exercise, resilience

## Abstract

Psychological crisis coping is an important component of college students' mental health adaptation, yet the psychological mechanisms through which physical exercise is associated with crisis coping remain insufficiently understood. Clarifying these mechanisms may help identify practical pathways for university-based mental health promotion. The present study examined the association between physical exercise and crisis coping among college students, with particular attention to the mediating roles of resilience and emotion regulation strategies, including cognitive reappraisal and expressive suppression. A convenience sampling method was employed, and data were collected via the Wenjuanxing platform from first- to third-year undergraduates, yielding 1,026 valid responses (60.04% male; mean age = 20.28 years). Structural equation modeling and bootstrap analysis were used to test the hypothesized relationships. The results showed that physical exercise was positively associated with crisis coping both directly and indirectly through multiple mediating pathways. Resilience emerged as a key mediator, and cognitive reappraisal was further associated with the indirect pathway. A significant chain mediation pathway—physical exercise → resilience → cognitive reappraisal → crisis coping—was also identified. These findings contribute to a better understanding of the psychological processes linking physical exercise to crisis coping and provide practical implications for integrating physical exercise into university mental health interventions.

## Introduction

A psychological crisis refers to a temporary disruption in psychological functioning that occurs when individuals face major stressors (Caplan, 1964). It is widely regarded as a critical stage for early intervention in the development of mental health problems (James et al., 2025). During the university period, students are exposed to multiple stressors, making it a particularly vulnerable stage for psychological difficulties. Evidence indicates that a substantial proportion of college students experience severe anxiety and psychological distress, underscoring the importance of crisis coping (CC) capacity in this population.

Although traditional intervention approaches—such as psychological counseling, family support, and mental health education—have been shown to be effective to some extent, they often face practical limitations, including limited resources, poor accessibility, and low acceptance among individuals (Gulliver et al., 2010). As a result, researchers have increasingly explored more economical, scalable, and non-specialist-dependent alternatives. Physical exercise, as a structured and low-cost behavioral strategy, has recently been recognized as an important factor associated with individuals' psychological adaptability. Empirical studies have shown that physical exercise is associated with improved emotional states, lower levels of stress, and higher self-efficacy (Biddle et al., 2019; Stubbs and Schuch, 2019). In a study of Chinese university students, Liao et al. (2023) found that regular physical exercise was associated with higher levels of subjective wellbeing and lower levels of depression and anxiety, suggesting that physical exercise may be an important psychological resource for college students' mental health. However, the specific processes through which physical exercise is associated with CC remain to be further clarified. In particular, although existing studies have identified resilience and emotion regulation (ER) as important psychological factors related to physical exercise, these factors have typically been examined in isolation or within simple mediation frameworks. Few studies have simultaneously examined the distinct roles of specific ER strategies—such as cognitive reappraisal (CR) and expressive suppression (ES)—together with resilience within a unified model of CC. Moreover, the potential sequential pathway linking these variables has not been systematically tested. In the present study, the term “physical exercise” refers to structured, planned, and repetitive bodily activities performed with the purpose of improving or maintaining physical fitness and psychological wellbeing. Compared with the broader concept of physical activity, which encompasses all forms of bodily movement, physical exercise specifically denotes purposeful and organized behaviors characterized by identifiable intensity, duration, and frequency (Zhang et al., 2025). This conceptual distinction is essential for the present study, as the measurement instrument (PARS-3) primarily captures structured exercise engagement rather than incidental physical movement.

ER is considered one of the core psychological processes underpinning individual psychological adaptation and may be associated with the quality of coping during stressful events (Gross, 1998). Among the various ER strategies, CR and ES are widely recognized as two prototypical forms (Gross and John, 2003). CR involves reframing the meaning of an emotional event to reduce its emotional impact and is generally regarded as a functional, adaptive strategy. In contrast, ES involves inhibiting the outward expression of emotions to maintain behavioral control, but is often associated with emotional exhaustion and the accumulation of negative affect. Previous research has shown that physical exercise is associated with a greater tendency to use CR and with a lower reliance on ES, which may, in turn, be related to overall ER capacity (Liao et al., 2023; Chacón-Cuberos et al., 2019). ER not only relates to stress responses but may also play an important role in linking behavioral factors, such as physical exercise, to ER and psychological adaptation.

Meanwhile, resilience, defined as an individual's ability to maintain psychological balance and achieve restorative growth in the face of adversity, plays an important role in coping with psychological crises (Masten, 2001). Individuals with high levels of resilience typically exhibit stronger ER, problem-solving, and social adaptation capabilities, and may demonstrate better recovery and coping when confronted with crises (Connor and Davidson, 2003). Physical exercise has also been associated with resilience, through factors such as positive emotions, self-efficacy, self-esteem, and stress tolerance (Silverman and Deuster, 2014; Xu et al., 2021). Thus, resilience may function not only as a mediating variable in the relationship between physical exercise and CC, but also as a potential bridge linking ER and CC. Previous literature has suggested a close and bidirectional relationship between resilience and ER. While some studies indicate that ER contributes to the development of resilience, other research suggests that resilience, as a relatively stable psychological resource, may be associated with the use of adaptive ER strategies (Tugade and Fredrickson, 2004; Bluth and Blanton, 2014). In the present study, we adopt the perspective that resilience, as a relatively stable psychological resource, may precede and be associated with the use of ER strategies, and we therefore specify the pathway from resilience to ER in the proposed model. This specification is theoretically grounded in the view that resilience reflects an individual's relatively stable adaptive capacity, which may provide the psychological foundation for the flexible and effective use of ER strategies under stress. This specification allows for a clearer examination of how internal psychological resources may be reflected in adaptive emotional responses in the context of CC. The developmental “resilience-building model” suggests that positive external stimuli (e.g., physical exercise) may be associated with internal psychological resources, which may, in turn, relate to adaptive emotional responses. However, existing studies have primarily examined physical exercise, resilience, and ER independently or within simple mediation frameworks. CC, as a complex psychological process, likely involves multiple processes operating in sequence rather than in isolation. From this perspective, a sequential pathway can be proposed in which physical exercise, as a positive behavioral input, may be associated with resilience as a relatively stable psychological resource, which may, in turn, relate to the use of adaptive ER strategies, and may further be associated with differences in CC. This stepwise conceptualization from external behavior to internal resources and then to regulatory processes provides a coherent theoretical basis for the proposed chain mediation model. To date, limited research has integrated these variables into a unified framework to explain how physical exercise is associated with CC. Therefore, the sequential roles of resilience and ER in this relationship remain insufficiently explored.

Therefore, the present study aims to construct a chain mediation model—(physical exercise → resilience → ER: CR / ES → CC)—based on a college student sample, in order to examine the potential pathways among these variables. Theoretically, this study advances existing research by integrating physical exercise, resilience, and distinct ER strategies into a unified chain mediation framework, thereby providing a more comprehensive understanding of the psychological processes underlying CC. Practically, the findings may offer potentially actionable and scalable implications for university mental health services, supporting the development of integrated “physical exercise + psychology” approaches. Specifically, this study addresses the following four core research questions:

*RQ1: Is physical exercise associated with students' CC through psychological factors such as ER and resilience? RQ2: Do different types of ER strategies play distinct roles in the relationship between physical exercise and CC? RQ3: Is resilience a key mediating factor in the relationship between physical exercise and CC? RQ4: Do ER and resilience jointly function as a chain mediation pathway linking physical exercise to CC?*

## Literature review

### The role of physical exercise in crisis coping

Physical exercise is not only beneficial for physical health but has also been widely recognized for its psychological regulatory functions. Systematic reviews have shown that physical exercise is associated with higher levels of positive emotions, self-efficacy, and social connectedness, while effectively reducing negative psychological states such as depression, anxiety, and stress responses (Biddle et al., 2019). These psychological benefits suggest that physical exercise may serve as a natural, low-cost, and scalable behavioral approach for promoting mental wellbeing among college students. From a theoretical perspective, CC refers to an individual's ability to mobilize emotional resources and engage in adaptive behaviors in response to stress or unexpected events. Its core mechanisms include ER, cognitive processing, and behavioral flexibility. Maher et al. (2014) found that Australian university students who engaged in moderate-to-vigorous physical exercise reported higher levels of psychological resilience and adaptability in the face of academic stress and interpersonal conflict. Similarly, Pasco et al. (2011) noted that physical exercise is associated with lower levels of stress responses and emotional crises through neurophysiological mechanisms, including cortisol regulation and the activation of dopamine and serotonin systems. However, most existing studies have focused on the role of physical exercise in alleviating clinical symptoms such as depression or anxiety, while relatively few have addressed its impact on CC as a preclinical and preventive construct. Moreover, many of these studies have relied on cross-sectional designs, which limit causal interpretation, and have primarily examined single psychological outcomes rather than integrated coping processes. As a result, the broader applicability of these findings to complex constructs such as CC remains limited. Therefore, it is important to further examine how physical exercise may be associated with individuals' capacity to cope with crises through specific psychological mechanisms and to clarify its functional role in the psychological defense system.

*H1: Physical exercise is positively associated with CC*.

### The mediating role of resilience

Resilience refers to an individual's capacity to maintain psychological stability, respond effectively, and achieve functional recovery when facing significant stress, frustration, or adversity (Masten, 2001). As a core variable in the psychological defense system, resilience not only mitigates the negative impacts of stress but also enhances emotional regulation and behavioral flexibility in crisis situations (Connor and Davidson, 2003). Previous studies have demonstrated that physical exercise is an effective means to enhance resilience. Silverman and Deuster (2014) found that individuals who regularly engage in physical exercise tend to exhibit higher levels of emotional recovery, self-efficacy, and stress tolerance. In an empirical study, Al-Wardat et al. (2024) demonstrated that higher levels of physical exercise were associated with enhanced resilience and better emotional regulation, while also reducing symptoms of anxiety and psychological distress among university students. Furthermore, resilience may serve as a mediating variable linking physical exercise and CC. On the one hand, physical exercise provides individuals with positive emotional resources and psychological experiences. On the other hand, enhanced levels of resilience can strengthen individuals' recovery and adaptability in crises, thereby improving their overall CC quality. Nevertheless, existing studies have yielded mixed findings regarding the directionality between resilience and ER, and have rarely examined how resilience operates within a sequential mechanism linking behavior and coping outcomes.

*H2: Physical exercise is associated with CC through the mediating role of resilience*.

### The mediating role of emotion regulation

ER refers to the psychological mechanisms by which individuals control the generation, experience, and expression of emotions (Gross, 1998). Under high-pressure situations, ER ability directly affects the intensity and duration of stress responses and serves as a key indicator of psychological adaptability (Aldao et al., 2010). Among various ER strategies, CR and ES are the most frequently studied. The former involves reinterpreting the meaning of an event to reduce emotional responses and is regarded as a highly functional and positive strategy; the latter aims to suppress outward emotional expressions to maintain control but may increase internal tension and physiological stress (Gross and John, 2003). Numerous studies support the facilitating effect of physical exercise on ER ability. A meta-analysis by Ahn and Fedewa (2011) found that physical exercise interventions significantly improved CR skills among children and adolescents. In a study of Chinese college students, Mu et al. (2024) found in a large-scale study involving 5,430 college students that physical exercise significantly enhanced self-efficacy, which in turn fully mediated the relationship between physical exercise and ER capacity. Specifically, physical exercise promoted greater confidence, which increased the use of CR while reducing reliance on ES, thereby improving emotional adaptability. Therefore, ER—especially the type of strategy employed—may play a mediating role between physical exercise and CC, serving as an essential psychological mechanism explaining how physical exercise influences individuals' responses to crises. Importantly, these two strategies may exert differential effects on CC. CR, by promoting adaptive cognitive restructuring and emotional flexibility, is more likely to facilitate effective coping responses. In contrast, ES, which primarily involves inhibiting emotional expression, may limit emotional processing and reduce coping effectiveness under stress. However, prior research has often examined ER in isolation, without considering its interaction with broader psychological resources such as resilience. In addition, the differential roles of specific strategies (e.g., CR vs. ES) in complex coping contexts remain insufficiently explored.

*H3a: Physical exercise is associated with CC through the mediating role of CR*.

*H3b: Physical exercise is associated with CC through the mediating role of ES*.

### The chain mediating roles of emotion regulation and resilience

In recent years, increasing attention has been paid to the dynamic relationship between ER and resilience. Tugade and Fredrickson proposed that effective ER enables individuals to recover quickly from negative emotional experiences, serving as a foundational mechanism for the development of resilience (Tugade and Fredrickson, 2004). Conversely, high levels of resilience enhance the stability with which individuals cope with emotional disturbances, suggesting a possible bidirectional and synergistic relationship. From a developmental psychology perspective, in Kumpfer's resilience framework model, suggested that positive external stimuli—such as physical exercise—can activate ER systems, thereby fostering the development of internal resilience mechanisms (Kumpfer, 1999). This chain pathway has been supported by several empirical studies among youth and university populations. For example, Renati et al. (2023) found that ER predicted university students' levels of resilience and partially mediated the relationship between physical exercise and psychological wellbeing. Similarly, Klosowska et al. (2020) demonstrated that under stress conditions, ER influenced adolescents' stress responses and subjective wellbeing indirectly through resilience. Taken together, there is strong justification to hypothesize that physical exercise may enhance individuals' use of ER strategies, which in turn strengthen their resilience, and ultimately improve their CC abilities. This sequential mediating mechanism offers a comprehensive explanation of how physical exercise contributes to psychological adaptation in high-stress contexts. Based on the theoretical perspective that resilience represents a relatively stable psychological resource that precedes and facilitates the use of ER strategies, we further propose a sequential mediation pathway in which resilience influences ER, which in turn affects CC.

*H4a: Physical exercise is associated with CC through a sequential mediation pathway involving resilience and ES*.

*H4b: Physical exercise is associated with CC through a sequential mediation pathway involving resilience and CR*.

### Proposed research model

**Figure 1:**
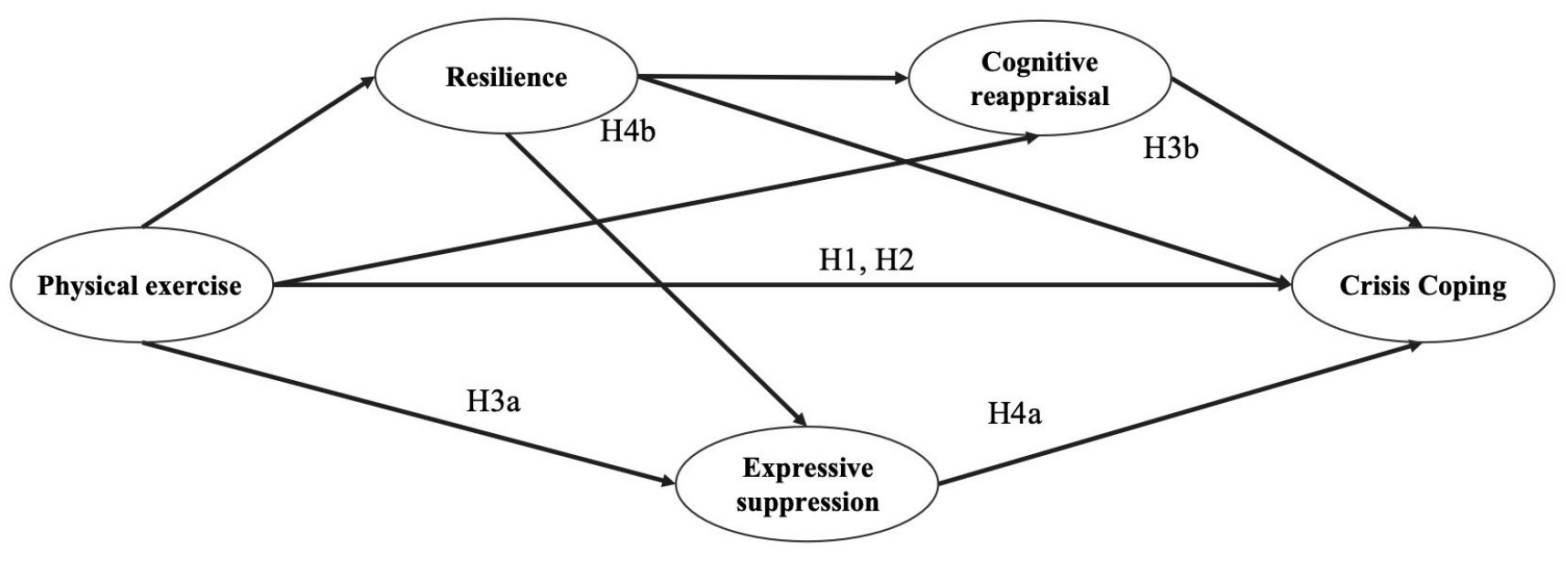
Hypothetical model of the relationship between physical exercise, resilience, emotion regulation, and crisis coping.

Based on these hypotheses, a research model was developed, as shown in Figure 1. Drawing upon existing literature, the present study constructed a chain mediation model to examine how physical exercise is associated with CC among university students through ER strategies and resilience. Specifically, the model hypothesizes that physical exercise has a direct positive effect on CC (H1), and also exerts an indirect effect via enhanced resilience (H2). In addition, two key ER strategies—CR and ES—are incorporated into the mediation pathway, each playing a partial mediating role (H3a, H3b). Furthermore, the model proposes that physical exercise is associated with resilience, which in turn is related to ER strategies, which in turn affects CR (H4a) or ES (H4b), ultimately contributing to improved CC. This model aims to uncover the underlying psychological mechanisms linking physical exercise and CC, and to provide both theoretical rationale and empirical evidence for the physical exercise—psychological regulation—CC pathway.

## Methods

This study was approved by the Ethics Committee of Nantong University (Ethical Approval No.: TD-2024-109), and informed consent was obtained from all participants.

### Participants

A convenience sampling approach was employed in this study. The survey was administered via a link generated on the Wenjuanxing platform (https://www.wjx.cn) and disseminated through popular Chinese social media channels, including WeChat and QQ, targeting universities located in Jiangsu, Anhui, and Liaoning provinces. To avoid potential disruptions due to graduation-related commitments, the sample was limited to first- through third-year undergraduate students. Data collection was carried out over a 4-week period, from April 16 to May 16, 2025. A total of 1,157 responses were initially received. After excluding submissions that met any of the following criteria—(1) more than 30% of items left unanswered, (2) missing gender or age information, or (3) responses that exhibited uniform patterns indicative of low response quality-−1,026 valid questionnaires were retained for analysis. Of the final sample, 616 participants were male (60.04%) and 410 were female (39.96%). Participants ranged in age from 17 to 23 years, with an average age of 20.28 years (SD = 1.53).

### Measures

#### Physical exercise

In this study, the Physical Activity Rating Scale (PARS-3), revised by Liang (1994), was employed to assess students' levels of physical exercise. Although the original scale refers to “physical activity,” it primarily captures structured physical exercise behaviors based on intensity, duration, and frequency. Therefore, in the present study, the term “physical exercise” is used to more accurately reflect the nature of the construct being measured. The scale comprises three items that measure the intensity (e.g., “What is the usual intensity of your physical exercise?”), duration (e.g., “How long does each session of physical exercise last?”), and frequency (e.g., “How often do you engage in physical exercise per month/week?”) of physical exercise. Each item provides five response options, rated on a scale from 1 to 5. For example, the options for physical exercise frequency range from “less than once a month” (one point) to “daily” (five points). The total score is calculated using the following formula: Score = Intensity × (Duration – 1) × Frequency. The score ranges from 0 to 100, with physical exercise levels categorized into three groups: low ( ≤ 19), moderate (20–42), and high (≥43). In the present study, the PARS-3 demonstrated good internal consistency, with a Cronbach's α coefficient of 0.798.

#### Resilience

Resilience was assessed using the Adolescent Resilience Scale developed by Hu and Gan (2008). This scale consists of 27 items and measures five core dimensions: Goal Focus, Interpersonal Assistance, Family Support, Emotional Control, and Positive Cognition. Each item is rated on a five-point Likert scale, with higher scores indicating a higher level of psychological resilience. In the present study, the scale demonstrated excellent internal consistency, with a Cronbach's α coefficient of 0.948.

#### Crisis coping

This study adopted a subscale from the College Students' Stress Response Questionnaire developed by Duan (2006), which assesses college students' coping strategies in response to psychological crises. The scale consists of 32 items and covers five dimensions: Problem Solving, Fantasy Thinking, Avoidance, Self-Blame, and Rationalization. It evaluates the specific strategies individuals use when facing psychological crisis situations. All items are rated on a five-point Likert scale, with higher scores indicating more frequent use of the corresponding strategy. In the present study, the scale demonstrated high internal consistency, with a Cronbach's α coefficient of 0.946, indicating good reliability and stability of the instrument.

#### Emotion regulation

To evaluate participants' emotion regulation strategies, this study utilized the Emotion Regulation Questionnaire (ERQ) developed byGross and John (2003). The Chinese version, revised by Wang et al. (2007), has been widely applied in adolescent populations in China and has shown satisfactory psychometric properties. This instrument has been culturally adapted and validated in Chinese samples, supporting its applicability in the present study. The ERQ comprises two subscales: cognitive reappraisal and expressive suppression, each containing seven items. Cognitive reappraisal reflects the individual's tendency to reinterpret emotional events to alter their emotional impact. ES captures the extent to which individuals consciously inhibit emotional expressions after emotional arousal. All items are rated on a seven-point Likert scale, ranging from 1 (strongly disagree) to 7 (strongly agree), with higher scores representing greater use of the corresponding strategy. In the current sample, the internal consistency of the ERQ was found to be high, with Cronbach's alpha coefficients of 0.875 for the cognitive reappraisal subscale and 0.884 for the expressive suppression subscale.

### Measurement model and validity assessment

To evaluate the measurement properties of the constructs, confirmatory factor analysis (CFA) was conducted using Amos 26.0. CFA was conducted prior to the structural equation modeling (SEM) analysis to ensure the adequacy of the measurement model. The adequacy of the measurement model was assessed based on standardized factor loadings, composite reliability (CR), and average variance extracted (AVE). Standard criteria were applied, with factor loadings above 0.70, CR values above 0.70, and AVE values above 0.50 considered indicative of acceptable convergent validity.

The CFA results (see Table 1) indicated that all standardized factor loadings exceeded 0.75, suggesting good indicator reliability. For the CC construct, AVE values ranged from 0.626 to 0.721 and CR values from 0.870 to 0.912. For the resilience construct, AVE values ranged from 0.646 to 0.738 and CR values from 0.879 to 0.944. For emotion regulation, the AVE values for CR and ES were 0.628 and 0.614, respectively, while the corresponding CR values were 0.922 and 0.918. Overall, these results support the reliability and convergent validity of the measurement model.

**Table 1:** Reliability and validity testing of the scale.

	Variable	Factor loading	AVE	CR
CC	Problem solving	0.841, 0.858, 0.805, 0.832	0.696	0.901
	Fantasy thinking	0.825, 0.829, 0.844, 0.804	0.682	0.895
	Avoidance	0.800, 0.837, 0.838, 0.812	0.676	0.893
	Self-blame	0.773, 0.790, 0.778, 0.823	0.626	0.870
	Rationalization	0.847, 0.877, 0.844, 0.827	0.721	0.912
Resilience	Emotional control	0.773, 0.790, 0.778, 0.823	0.626	0.870
	Goal focus	0.872, 0.840, 0.869, 0.863, 0.832	0.732	0.932
	Interpersonal assistance	0.866, 0.899, 0.843, 0.844, 0.864, 0.837	0.738	0.944
	Family support	0.843, 0.866, 0.872, 0.778, 0.841, 0.829	0.703	0.934
	Positive cognition	0.803, 0.820, 0.815, 0.776	0.646	0.879
ER	Cognitive reappraisal	0.765, 0.807, 0.764, 0.778, 0.793, 0.829, 0.807	0.628	0.922
	Expressive suppression	0.775, 0.801, 0.791, 0.796, 0.782, 0.782, 0.758	0.614	0.918

CC, crisis coping; ER, emotion regulation.

### Common method bias

To assess the potential impact of common method bias (CMB) due to the use of self-reported questionnaires, both procedural and statistical approaches were employed. Procedurally, the survey items were carefully adapted to ensure cultural and linguistic appropriateness for Chinese college students, and participants were informed of the anonymity and confidentiality of their responses to reduce evaluation apprehension and social desirability bias. Statistically, Harman's single-factor test was conducted using unrotated exploratory factor analysis. The first factor accounted for 21.351% of the total variance, which is below the commonly accepted threshold of 40%, suggesting that CMB is unlikely to have substantially influenced the findings (Zhou and Long, 2004; Wen and Ye, 2014). In addition, although Harman's test has certain limitations, the use of both procedural remedies and statistical diagnostics helps to mitigate, although not entirely eliminate, the potential impact of CMB. However, it should be noted that Harman's single-factor test has certain limitations. Therefore, the results should be interpreted with caution.

### Statistical analysis

Prior to analysis, the distribution of the data was examined using skewness and kurtosis indices. The results indicated that several variables showed deviations from normality. Therefore, non-parametric tests were used for group comparisons. However, Pearson correlation analysis was retained for examining relationships among variables, as it is considered robust to moderate violations of normality, particularly in large samples. SEM was conducted using Amos 26.0 software. The sample size (*N* = 1,026) was considered adequate for SEM analysis, meeting commonly recommended criteria for model estimation in behavioral research. Statistical significance was determined at a *p*-value of less than 0.05. To test the mediating effects of ER and resilience, the bias-corrected bootstrap method with 5,000 resamples was applied, and 95% confidence intervals were reported.

## Results

### Analysis of group differences in physical exercise, resilience, emotion regulation, and crisis coping

This section presents the results of group difference analyses for physical exercise, resilience, ER, and CC. To examine potential group differences in physical exercise, resilience, ER, and CC, Mann–Whitney *U*-tests (for gender) and Kruskal–Wallis *H*-tests (for age) were conducted due to non-normality of the data distribution (see Table 2). The results revealed a statistically significant gender difference in physical exercise level (*Z* = −2.986, *p* = 0.003), with male participants (*M* = 37.03, SD = 31.89) showing higher levels than females (*M* = 30.34, SD = 27.95). CC also exhibited a significant gender difference (*Z* = −2.445, *p* = 0.014), where males (*M* = 3.50, SD = 0.75) scored slightly lower in average than females (*M* = 3.56, SD = 0.83). However, gender differences in resilience (*Z* = −1.839, *p* = 0.066), CR (*Z* = −0.860, *p* = 0.390), and ES (*Z* = −1.251, *p* = 0.211) were not statistically significant. Regarding age, the Kruskal–Wallis *H*-test results showed no significant differences in physical exercise (*H* = 3.321, *p* = 0.768), resilience (*H* = 7.064, *p* = 0.315), CR (*H* = 2.406, *p* = 0.879), or ES (*H* = 1.236, *p* = 0.975). No significant difference was observed in CC (*H* = 4.852, *p* = 0.563), it did not reach statistical significance. Overall, gender differences were only observed in physical exercise and CC, while age showed no significant group effects across the measured variables.

**Table 2:** Mann–Whitney *U*-test for gender and Kruskal–Wallis *H*-test for Age groups (*M* ± SD).

Variables	Physical exercise	CC	resilience	ER	
				CR	ES
Mann–Whitney *U*	112,423.500	114,914.000	117,733.500	122,284.000	120,469.000
Wilcoxon *W*	196,678.500	199,169.000	201,988.500	206,539.000	310,505.000
*Z*	−2.986	−2.445	−1.839	−0.86	−1.251
Asymptotic Sig. (two-tailed)	0.003	0.014	0.066	0.390	0.211
Kruskal–Wallis *H*	3.321	4.852	7.064	2.406	1.236
Df	6	6	6	6	6
Sig.	0.768	0.563	0.315	0.879	0.975
Male	37.03 ± 31.89	3.50 ± 0.75	3.32 ± 0.85	4.50 ± 1.40	4.76 ± 1.32
Female	30.34 ± 27.95	3.56 ± 0.83	3.21 ± 0.87	4.40 ± 1.44	4.82 ± 1.39
17	36.17 ± 34.22	3.51 ± 0.77	3.46 ± 0.93	4.43 ± 1.43	4.70 ± 1.53
18	39.32 ± 31.18	3.52 ± 0.71	3.33 ± 0.79	4.60 ± 1.39	4.77 ± 1.33
19	36.46 ± 30.07	3.47 ± 0.77	3.18 ± 0.90	4.52 ± 1.42	4.77 ± 1.27
20	33.89 ± 30.13	3.47 ± 0.75	3.26 ± 0.86	4.46 ± 1.39	4.83 ± 1.29
21	33.51 ± 30.92	3.38 ± 0.80	3.32 ± 0.85	4.45 ± 1.42	4.75 ± 1.45
22	32.07 ± 30.13	3.37 ± 0.79	3.24 ± 0.81	4.35 ± 1.37	4.87 ± 1.37
23	32.05 ± 34.38	3.48 ± 0.88	3.33 ± 0.84	4.36 ± 1.54	4.75 ± 1.28

CC, crisis coping; ER, emotion regulation; CR, cognitive reappraisal; ES, expressive suppression.

### Descriptive statistics and correlation analysis

This section reports the descriptive statistics and correlation analysis among the main study variables (Table 3). The results indicated that physical exercise (*M* = 34.35, SD = 30.53) was significantly and positively correlated with CC (*r* = 0.471, *p* < 0.01), resilience (*r* = 0.442, *p* < 0.01), and CR (*r* = 0.370, *p* < 0.01), and negatively correlated with ES (*r* = −0.383, *p* < 0.01). CC (*M* = 4.45, SD = 1.41) was significantly associated with resilience (*r* = 0.567, *p* < 0.01), CR (*r* = 0.478, *p* < 0.01), and ES (*r* = −0.381, *p* < 0.01). Furthermore, resilience (*M* = 4.78, SD = 1.34) showed positive correlations with CR (*r* = 0.437, *p* < 0.01) and negative correlations with ES (*r* = −0.339, *p* < 0.01). Finally, CR (*M* = 3.27, SD = 0.85) and ES (*M* = 3.44, SD = 0.78) were negatively correlated (*r* = −0.472, *p* < 0.01). All correlations were statistically significant at the *p* < 0.01 level. Although some correlations were moderate to relatively high, all coefficients were below the commonly accepted threshold (e.g., *r* < 0.80), suggesting that multicollinearity was not a serious concern in the present study.

**Table 3:** Mean and correlation between variables.

Variables	*M*	SD	Physical exercise	CC	Resilience	CR	ES
Physical exercise	34.35	30.53	1				
CC	4.45	1.41	0.471^**^	1			
Resilience	4.78	1.34	0.442^**^	0.567^**^	1		
CR	3.27	0.85	0.370^**^	0.478^**^	0.437^**^	1	
ES	3.44	0.78	−0.383^**^	−0.381^**^	−0.339^**^	−0.472^**^	1

CC, crisis coping; CR, cognitive reappraisal; ES, expressive suppression.

^**^p < 0.01.

### Model construction and hypothesis testing

This section evaluates the overall fit of the structural equation model. The structural equation model demonstrated an excellent overall model fit (see Table 4). The chi-square to degrees of freedom ratio (χ^2^/df) was 1.211, which is well-below the recommended threshold of 3.000. Other key fit indices also indicated a good model fit: RMSEA = 0.014 (<0.080), CFI = 0.986, GFI = 0.925, AGFI = 0.920, NFI = 0.924, and IFI = 0.986—all exceeding the commonly accepted cutoff value of 0.900. These fit indices indicate an acceptable model fit. These fit indices indicate that the proposed model adequately represents the observed data. Importantly, the model structure was specified based on theoretical considerations regarding the relationships among physical exercise, resilience, ER, and CC, rather than purely data-driven modifications.

**Table 4:** Model fit indices.

Evaluation indicators	Model fit value	Judgment standard
χ^2^/df	1.211	<3.000, good fit
RMSEA	0.014	<0.080, good fit
CFI	0.986	>0.900, good fit
GFI	0.925	>0.900, good fit
AGFI	0.920	>0.900, good fit
NFI	0.924	>0.900, good fit
IFI	0.986	>0.900, good fit

**Figure 2:**
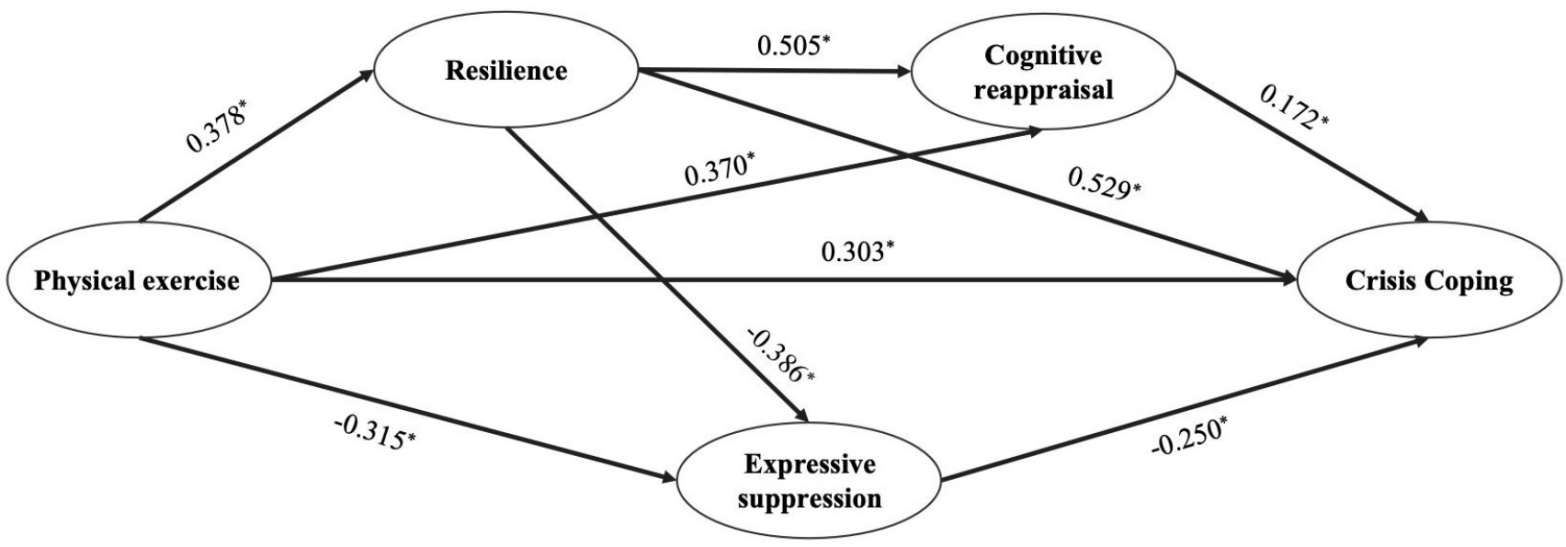
Path analysis results of physical exercise, resilience, emotion regulation, crisis coping. The coefficients in the graphs are standardized; ^*^p < 0.05.

This section presents the results of the structural path analysis. Figure 2 presents the standardized path coefficients of the structural model. Physical exercise significantly predicted resilience (β = 0.378, *p* < 0.05), CR (β = 0.370, *p* < 0.05), ES (β = −0.315, *p* < 0.05), and CC (β = 0.303, *p* < 0.05). In turn, resilience significantly predicted CR (β = 0.505, *p* < 0.05), ES (β = −0.386, *p* < 0.05), and CC (β = 0.529, *p* < 0.05). Moreover, CR (β = 0.172, *p* < 0.05) and ES (β = −0.250, *p* < 0.05) were also significant predictors of CC. All structural paths were statistically significant (*p* < 0.05). Overall, the magnitude of the path coefficients suggests that the relationships among physical exercise, resilience, ER, and CC are of moderate practical significance.

Notably, the path from resilience to CC showed a relatively strong effect (β = 0.529), indicating a relatively stronger association compared to other paths. In contrast, the effects associated with ER strategies, although statistically significant, were comparatively smaller, indicating that these mechanisms are relatively weaker compared to other pathways.

### Analysis of the mediating effect

This section examines the mediating effects of resilience and ER in the relationship between physical exercise and CC using a bias-corrected bootstrap method. As shown in Table 5, the total indirect effect of physical exercise on CC was statistically significant (β = 0.267, SE = 0.025, 95% CI [0.222, 0.320]), accounting for 62.57% of the total effect. The direct effect remained significant (β = 0.160, SE = 0.037, 95% CI [0.088, 0.232]), suggesting partial mediation. Among the specific indirect pathways, the effect via resilience alone was the strongest (β = 0.164, SE = 0.023, 95% CI [0.122, 0.214]), explaining 38.41% of the total effect. The paths through CR (β = 0.038, SE = 0.010, 95% CI [0.020, 0.060]) and ES (β = 0.018, SE = 0.009, 95% CI [0.002, 0.038]) were also significant but smaller in magnitude. Two chain mediation effects were found to be significant: physical exercise → resilience → CR → CC (β = 0.039, SE = 0.008, 95% CI [0.025, 0.058]), and physical exercise → resilience → ES → CC (β = 0.009, SE = 0.004, 95% CI [0.001, 0.019]). The mediation analysis results showed that both resilience and ER were significantly involved in the association between physical exercise and CC.

**Table 5:** Chain mediated path effect test for resilience and emotion regulation.

Path	Effect value	Bootstrap SE	Bootstrap 95%CI		Proportion of total effect (%)
			Lower	Upper	
Physical exercise → Resilience → CC	0.164	0.023	0.122	0.214	38.41%
Physical exercise → CR → CC	0.038	0.010	0.020	0.060	8.90%
Physical exercise → ES → CC	0.018	0.009	0.002	0.038	4.21%
Physical exercise → Resilience → CR → CC	0.039	0.008	0.025	0.058	9.13%
Physical exercise → Resilience → ES → CC	0.009	0.004	0.001	0.019	2.11%
Total indirect effect	0.267	0.025	0.222	0.320	62.57%
Direct effect	0.160	0.037	0.088	0.232	37.47%
Total effect	0.427	0.027	0.374	0.480	100.00%

CC, crisis coping; CR, cognitive reappraisal; ES, expressive suppression.

The results indicate that physical exercise exerts both direct and indirect effects on CC. The direct effect reflects the independent contribution of physical exercise to coping outcomes, whereas the indirect effects indicate that this relationship is partially transmitted through resilience and ER. These findings suggest that resilience and ER function as important pathways linking physical exercise to CC, supporting the proposed mediation model.

## Discussion

From an interdisciplinary perspective, the present findings can be understood through the integration of sport psychology and crisis intervention frameworks. Physical exercise, as emphasized in sport psychology, is considered a behavioral factor that is associated with emotional and cognitive functioning. Meanwhile, from a crisis intervention perspective, resilience and ER represent core psychological mechanisms that determine individuals' responses to acute stress. The integration of these perspectives highlights that physical exercise may be understood not only as a health behavior but also as a preventive psychological factor associated with individuals' CC capacity.

### Group differences in physical exercise, resilience, emotion regulation, and crisis coping

Consistent with previous research, female students demonstrated stronger CC abilities than male students. For example, a study found that female college students were more likely to adopt coping strategies such as emotional expression and seeking support, whereas males were more inclined toward avoidance and suppression (Graves et al., 2021). These strategic preferences may be associated with stronger ER and recovery abilities during crises. Similarly, Urbón et al. (2025) found that female students scored significantly higher on emotional intelligence factors related to emotion awareness, expression, and regulation—psychological strengths closely associated with enhanced wellbeing and adaptive coping mechanisms. From a sociocultural perspective, these gender differences may reflect traditional gender role expectations. Males are often encouraged to suppress emotional expression and emphasize independence, whereas females are more likely to engage in emotional communication and seek social support, which may be associated with their relative advantage in coping with psychological stress. According to Masten's classical theory, these emotional and social advantages may form an important foundation for females‘ resilience (Masten, 2001). Secondly, in terms of ER strategies, females scored higher in CR, while males relied more on ES. This gender difference is supported by a meta-analysis by McRae et al. (2008). CR is considered a more adaptive strategy, allowing individuals to reduce negative emotions by altering their appraisal of events (Gross, 1998), whereas ES may intensify internal tension and physiological stress (Gross and John, 2003). These differences may be related to the observed differences in CC between females and males. Regarding grade-level differences, third-year students showed relatively lower scores in physical exercise frequency and resilience, possibly due to increased stress related to graduation, postgraduate entrance exams, or job searching. Previous research has reported a decrease in resilience over the course of students' training, particularly in areas such as ambiguity tolerance and resilience, suggesting that prolonged academic stress may be associated with the erosion of internal coping resources (Eley et al., 2022). Similarly, Zhang et al. (2021) found that both physical exercise motivation and participation declined across college years, which may be associated with lower levels of emotional adaptability and mental recovery over time. These findings appear to differ from the notion proposed by Connor and Davidson (2003) that resilience is derived from the accumulation of coping resources, suggesting that upper-year students may be at risk of resource depletion. Notably, although male students generally reported higher levels of physical exercise than females, they did not show superior performance in ER or CC. This finding indicates that the psychological benefits of physical exercise may not be solely related to activity frequency, but may also be related to the extent to which individuals experience adaptive emotional regulation and cognitive processing during physical exercise (Li, 2024).

In contrast, the sequential mediation pathway involving ES (physical exercise → resilience → ES → CC) showed a relatively small effect size. This finding indicates that ES may play a more limited role in linking physical exercise to CC compared to CR. It should be noted that the effect size of this pathway was relatively small and should therefore be interpreted with caution. One possible explanation is that ES is generally considered a less adaptive ER strategy. Unlike CR, which modifies the meaning of emotional events, ES primarily involves inhibiting emotional expression without addressing the underlying emotional experience. Accordingly, even when physical exercise is associated with higher levels of resilience, its indirect association with CC through ES may remain relatively weak. From a practical perspective, this finding further suggests that adaptive ER strategies, such as CR, may be more relevant in physical exercise-based contexts compared to suppression-based coping patterns.

In summary, the analysis of group differences not only reveals how psychological regulation mechanisms vary across demographic groups but also underscores the importance of designing interventions with sensitivity to gender and developmental stage. Future mental health promotion programs should be grounded in classical psychological mechanisms while also taking into account the stress ecology and behavioral patterns of contemporary college students, thereby informing the development of more targeted “physical exercise-psychology” integrated intervention approaches.

To enhance the practical applicability of these findings, a campus-based “physical exercise + psychological resilience” integrated intervention program may be considered. Such a program could potentially combine structured physical exercise sessions (e.g., moderate-intensity aerobic exercise, group sports, or traditional mind-body practices) with psychological skills training, including resilience-related components and ER strategies. For example, weekly intervention sessions could include guided physical exercise followed by brief cognitive-behavioral components, such as CR training, stress management techniques, and peer-based reflection or sharing activities. This integrated approach may be associated with improvements in physical fitness and the development of adaptive psychological resources, which may, in turn, be related to differences in CC. In addition, the program could be tailored to different student groups (e.g., by gender or academic year) to better address diverse coping patterns and stress contexts. Such context-sensitive and mechanism-based approaches may provide a potentially useful direction for supporting mental health among college students.

### The direct effect of physical exercise on crisis coping

This study further revealed a significant direct effect of physical exercise on college students' CC abilities. This finding suggests that the mediating variables included in the present study do not fully account for the association between physical exercise and CC. One possible explanation is that additional unmeasured mechanisms may contribute to this association. For example, physical exercise has been associated with higher levels of self-efficacy, social connectedness, and access to social support, all of which are important factors in psychological adaptation (Pan et al., 2025; Hu and Liu, 2025). These variables were not included in the current model and may partially account for the remaining direct effect. From this perspective, physical exercise may be related to CC through multiple pathways, including both the psychological mechanisms examined in this study and other social and cognitive processes. Future research is encouraged to incorporate a broader range of mediating variables to further clarify the underlying mechanisms linking physical exercise and CC.

### The mediating role of resilience

This study further supported the mediating role of resilience in the relationship between physical exercise and CC, supporting the hypothesized pathway that physical exercise is associated with CC through resilience as a mediating mechanism. This finding aligns with Masten's classic definition of resilience, which emphasizes the importance of individuals' internal psychological resources and adaptive capacity in achieving positive recovery when facing stress and challenges (Masten, 2001). It also corresponds with the Protective Factor Model proposed by Luthar et al. (2000) which posits that resilience, as a mediating resource variable, serves a buffering and transforming role between stress and adaptation. As a positive and accessible lifestyle behavior, physical exercise has been empirically associated with the development and maintenance of resilience through factors such as self-efficacy, positive emotions, and social connectedness (Silverman and Deuster, 2014). For instance, Trottier et al. (2021) found in an intervention study that college students who engaged in regular physical exercise showed significant improvements in dimensions of resilience such as self-acceptance, emotional regulation, and interpersonal adaptation. Shao et al. (2025) further argued that physical exercise could be associated with emotional recovery and cognitive flexibility, which may in turn be related to a more stable stress-resistance structure and greater coping capacity under adversity. It is worth noting that Connor and Davidson (2003) emphasized that the core of resilience lies in “functional recovery and goal orientation in the face of stress,” which is influenced by the dynamic interaction of emotional, cognitive, and behavioral systems. Physical exercise may be related to the activation of this system and may also be associated with its sustained functioning. In this context, resilience plays a bridging role in the current model: on the one hand, it receives the positive psychological resources activated by physical exercise; on the other hand, it functions as a core mechanism for mobilizing coping resources in crisis situations. In conclusion, the establishment of resilience as a mediating mechanism enriches the theoretical framework of how physical exercise is associated with psychological adaptation and may provide a useful reference for developing integrated intervention designs based on physical exercise-psychological resource co-activation in university settings. In practical terms, future interventions are recommended to incorporate resilience as a key target of physical exercise programs, thereby enhancing college students' capacity for autonomous recovery and emotional regulation under high-pressure conditions.

### The mediating roles of emotion regulation

This study further found that ER strategies played a significant mediating role in the relationship between physical exercise and CC, with CR and ES exhibiting positive and negative mediation effects, respectively. These results support the process model of ER proposed by Gross (1998), which emphasizes that the choice of ER strategy critically determines psychological outcomes in coping contexts. CR, as an antecedent-focused strategy, helps individuals reinterpret situations to reduce negative emotional experiences and is widely regarded as an adaptive regulation method (Gross and John, 2003). In contrast, ES, a response-focused strategy, may temporarily maintain emotional stability but often increases internal psychological burden and impairs social interactions, ultimately depleting individuals' coping resources (Hu et al., 2014). Physical exercise has been associated with differences in individuals' ER tendencies. Sheng et al. (2024), in a sample of college students, found that regular physical exercise was associated with a higher frequency of using CR and a lower reliance on ES. This may be due to the natural opportunities for emotional release and cognitive processing during physical exercise, which may, in turn, be associated with a greater tendency to adopt more positive, reconstruction-based regulation strategies in daily life. An fMRI study by Zhang et al. (2021) provided evidence relevant to this mechanism, revealing that physical exercise interventions were associated with greater prefrontal cortex activation related to CR, suggesting a neurobiological pathway through which physical exercise may be linked to adaptive ER. The findings of this study also indicated that CR not only mediated the association between physical exercise and CC but was also associated with individuals' cognitive flexibility and capacity for positive appraisal under stress, thereby indirectly strengthening their confidence and motivation to cope with crises. In contrast, the mediation pathway of ES was negative, suggesting that although commonly used, this strategy may be associated with less favorable psychological recovery outcomes and adaptive coping in the long term. Xu et al. (2024) found that college students who rely heavily on ES are more prone to emotional exhaustion and distorted crisis perception, underscoring the need for higher education interventions to promote alternatives to maladaptive ER strategies. In sum, ER may be considered a key psychological mechanism that bridges the link between physical exercise and CC, while also demonstrating the potential of physical exercise interventions to reshape emotion processing strategies. In future practice, it is recommended that ER training—especially the enhancement of CR ability—be embedded into physical exercise promotion programs to build an integrated “physical exercise-regulation-coping” intervention framework. Such a system would help college students develop a more stable and proactive psychological defense structure.

### The chain mediating roles of resilience and emotion regulation

This study further supported the proposed mechanism by which physical exercise is associated with college students' CC ability through a sequential pathway of resilience → ER, demonstrating a significant chain mediation effect. This finding suggests that physical exercise is not only directly associated with psychological coping resources but may also be related to CC through resilience and subsequent ER strategies. This result aligns with Kumpfer (1999)'s “transitional resilience model,” which posits that individuals' internal self-regulatory mechanisms are gradually activated following positive external stimuli—such as physical exercise—and subsequently influence their ER strategies and stress response systems. Empirical studies support this pathway. Cowdrey (2025) indicated that physical exercise was associated with adolescents' psychological adaptability and with their ability to remain stable under pressure and integrate coping resources. This pattern may, in turn, be associated with a greater tendency to adopt constructive strategies such as CR and a lower reliance on maladaptive approaches such as ES. Furthermore, Polizzi and Lynn (2021) found that college students with higher levels of resilience tend to possess stronger emotional insight and regulatory intentions, making them more likely to employ constructive ER strategies when facing challenges. These findings provide both theoretical justification and empirical evidence for the mechanism path of physical exercise → resilience → ER → CC. More specifically, this study found that the path from resilience to CR showed a positive mediating effect, whereas the path from resilience to ES showed a negative effect, further highlighting the functional differences between the two types of ER strategies within the chain mechanism. CR, as a proactive regulatory strategy, may exert its positive effect by leveraging the cognitive flexibility and recovery capacity conferred by resilience, enabling individuals to extract positive meaning from stressful events and reframe their evaluations (Gross and Thompson, 2007). This integrative use of psychological resources contributes to more efficient coping structures and may be associated with higher confidence and perceived effectiveness in crisis handling. In contrast, when individuals possess a certain level of resilience but still tend to suppress emotional expression, it may indicate cognitive or social expression difficulties, which hinder the externalization and effective use of internal resources (John and Gross, 2004). This phenomenon is particularly pronounced under high-stress conditions such as during the COVID-19 pandemic. Kuhlman et al. (2023) found that students with moderate levels of resilience were more likely to overuse ES strategies in high-pressure contexts, ultimately weakening their crisis regulation outcomes. These findings suggest that the chain mediation pathway is not automatically realized but depends on the type of ER strategy activated by resilience. Therefore, in university mental health contexts, merely focusing on students' resilience may be insufficient. It may also be important to consider how internal resilience is reflected in outward, adaptive ER behaviors, particularly among college populations whose emotional management capacities may still be developing.

## Conclusion

This study investigated the relationship between physical exercise and CC among college students, with a focus on the mediating roles of resilience and ER. The results revealed that physical exercise was not only associated with CC through a direct pathway but was also indirectly associated with CC via resilience, CR, and ES. Among these, resilience emerged as the most prominent mediator. In addition, two significant chain mediation pathways were identified. This study extends the understanding of the interaction between behavioral factors and psychological mechanisms, offering both theoretical implications and practical significance for mental health interventions.

### Limitations and future research directions

Given the cross-sectional design of this study, the findings should be interpreted as associations rather than causal relationships. Despite its contributions, this study has several limitations that warrant consideration. First, the cross-sectional design limits the ability to draw causal inferences between variables. Future research should consider longitudinal or experimental designs to further clarify the causal relationships among variables. Second, all data were collected through self-report questionnaires, which may introduce potential biases such as social desirability bias and CMB. Although Harman's single-factor test was conducted to assess CMB, the possibility of subjective bias cannot be fully ruled out. Future studies are encouraged to incorporate more objective measures, such as behavioral tracking or physiological indicators (e.g., heart rate variability), to enhance the robustness of the findings. Third, the sample was based on a convenience sampling approach and limited to college students from three provinces in China (Jiangsu, Anhui, and Liaoning), which may restrict the representativeness of the sample and limit the external validity and generalizability of the findings to other populations. Future research should adopt more diverse and representative sampling strategies across different regions, cultural contexts, and age groups. Finally, the current study did not examine potential moderating variables such as gender, stress intensity, or social support. In addition, although gender differences were observed in preliminary analyses, gender was not included as a covariate in the structural model, which may limit the precision of the estimates. Future research could include these factors to better understand the conditions under which physical exercise is associated with CC.

## Data Availability

The original contributions presented in the study are included in the article/Supplementary material, further inquiries can be directed to the corresponding author.
